# Prevention of bone dehiscence associated with orthodontic tooth movement by prophylactic injection of bone anabolic agents in mice

**DOI:** 10.1038/s41598-024-66617-6

**Published:** 2024-07-08

**Authors:** Jia Qi, Yoshiro Matsumoto, Cangyou Xie, Fatma Rashed, Takashi Ono, Kazuhiro Aoki

**Affiliations:** 1 Present Address: Department of Orthodontic Science, Graduate School of Medical and Dental Sciences, Tokyo Medical and Dental University (TMDU), Tokyo, Japan; 2 Department of Basic Oral Health Engineering, Graduate School of Medical and Dental Sciences, Tokyo Medical and Dental University (TMDU), Tokyo, Japan; 3 Department of Oral Pathology, Graduate School of Medical and Dental Sciences, Tokyo Medical and Dental University (TMDU), Tokyo, Japan; 4 Department of Oral Biology, Faculty of Dentistry, Damanhour University, Damanhour, 22511 Egypt

**Keywords:** Alveolar bone loss, Bone regeneration, OP3-4 peptide, Drug delivery systems, Orthodontic tooth movement, Bone remodelling, Biomaterials - proteins, Drug delivery

## Abstract

Although bone dehiscence may occur during orthodontic tooth movement into the narrow alveolar ridge, a non-invasive prevention method is yet to be fully established. We show for the first time prevention of bone dehiscence associated with orthodontic tooth movement by prophylactic injection of bone anabolic agents in mice. In this study, we established a bone dehiscence mouse model by applying force application and used the granular type of scaffold materials encapsulated with bone morphogenetic protein (BMP)-2 and OP3-4, the receptor activator of NF-κB ligand (RANKL)-binding peptide, for the prophylactic injection to the alveolar bone. In vivo micro-computed tomography revealed bone dehiscence with decreased buccal alveolar bone thickness and height after force application, whereas no bone dehiscence was observed with the prophylactic injection after force application, and alveolar bone thickness and height were kept at similar levels as those in the control group. Bone histomorphometry analyses revealed that both bone formation and resorption parameters were significantly higher in the injection with force application group than in the force application without the prophylactic injection group. These findings suggest that the prophylactic local delivery of bone anabolic reagents can prevent bone dehiscence with increased bone remodelling activity.

## Introduction

Insufficient alveolar bone volume is a common problem encountered in dental treatments, including periodontal disease treatment, prosthetic reconstruction, tooth transplantation treatment, and orthodontic treatment^1^. In orthodontic treatment, the thickness of the alveolar bone defines one of the boundaries of the tooth movement. Tooth movement in a constricted alveolar ridge, including molar buccal expansion, space closure of extraction or missing sites in an atrophic alveolar ridge (AAR), and incisor labio-lingual movements, may cause undesirable collateral impacts such as bone dehiscence, gingival recession, root resorption and reduction in tooth movement rate^2–4^. The presence of bone dehiscence has been proven to jeopardise the aesthetic appearance of the periodontal tissue and negatively affect the long-term stability of orthodontic treatment^5–8^.

During dental arch expansion, as the roots move into the thin buccal alveolar bone plate by force, there could be perforation of the cortical plate. Previous clinical studies using cone-beam computed tomography (CBCT) reported that rapid maxillary expansion induces bone dehiscence, along with a decrease in the buccal alveolar bone thickness and height of the supporting teeth^9,10^. Another clinical challenge is the space closure of edentulous regions in the AAR. AAR often manifests as a pronounced buccolingual collapse and crestal bone loss. To achieve space closure in the AAR, reshaping of the cortical bone is required^11,12^. Studies using CBCT images confirmed that bone dehiscence increased after tooth movement through the AAR, mainly in the buccal plate^13^. Tooth movement through the AAR also results in the loss of periodontal support^14,15^. Regarding the labio-lingual movements of the mandibular incisors, a decrease in alveolar bone thickness and root perforations of the lingual cortical plates are often detected^16,17^.

To overcome these anatomical restrictions against tooth movement, various studies have made efforts to obtain alveolar bone augmentation and prevent bone dehiscence^18–21^. However, these invasive surgical procedures using bone grafting methods or osteoperforation are often associated with the risk of harvesting healthy bone, potential infections, and postoperative pain^22,23^. In addition, it is also reported that clinical bone substitutes such as Bio-Oss grafts could slow down orthodontic tooth movement^24^.

Alternative minimally invasive approaches for alveolar bone augmentation have been actively investigated to mitigate the collateral effects of surgical procedures. Injection of injectable growth factors using gelatin hydrogel as a carrier is considered a safe and feasible method for tissue regeneration. The injection method was shown to reduce the operating time and minimise damage to surrounding tissue^25^. Bone morphogenetic protein (BMP)-2 is a strong growth factor clinically used to induce local bone formation^26,27^. On the other hand, a new anabolic reagent, OP3-4 peptide, which is the receptor activator of NF-κB ligand (RANKL)-binding peptide, is known to inhibit bone resorption and enhance BMP-2-induced local bone formation in vitro and in vivo^28–30^. We developed an injection technique that allowed the incorporation of BMP-2 and OP3-4 peptide into a granular type of gelatin hydrogel carrier to achieve local alveolar bone augmentation in a mouse model^31,32^. However, the preventive effect of this injection method on bone dehiscence has not been investigated, since a mouse model of bone dehiscence induced by tooth-movement has not been established yet.

In this study, we established a novel mouse model of FA-induced alveolar bone dehiscence. Using this model and bone histomorphometric measurements, we showed the preventive effect of the minimally invasive anabolic injection of BMP-2 and OP3-4 peptide on bone dehiscence induced by force application and an increase of tooth movement in mice with high bone remodelling activities.

## Results

### Bone dehiscence appeared during force application

**Figure 1:**
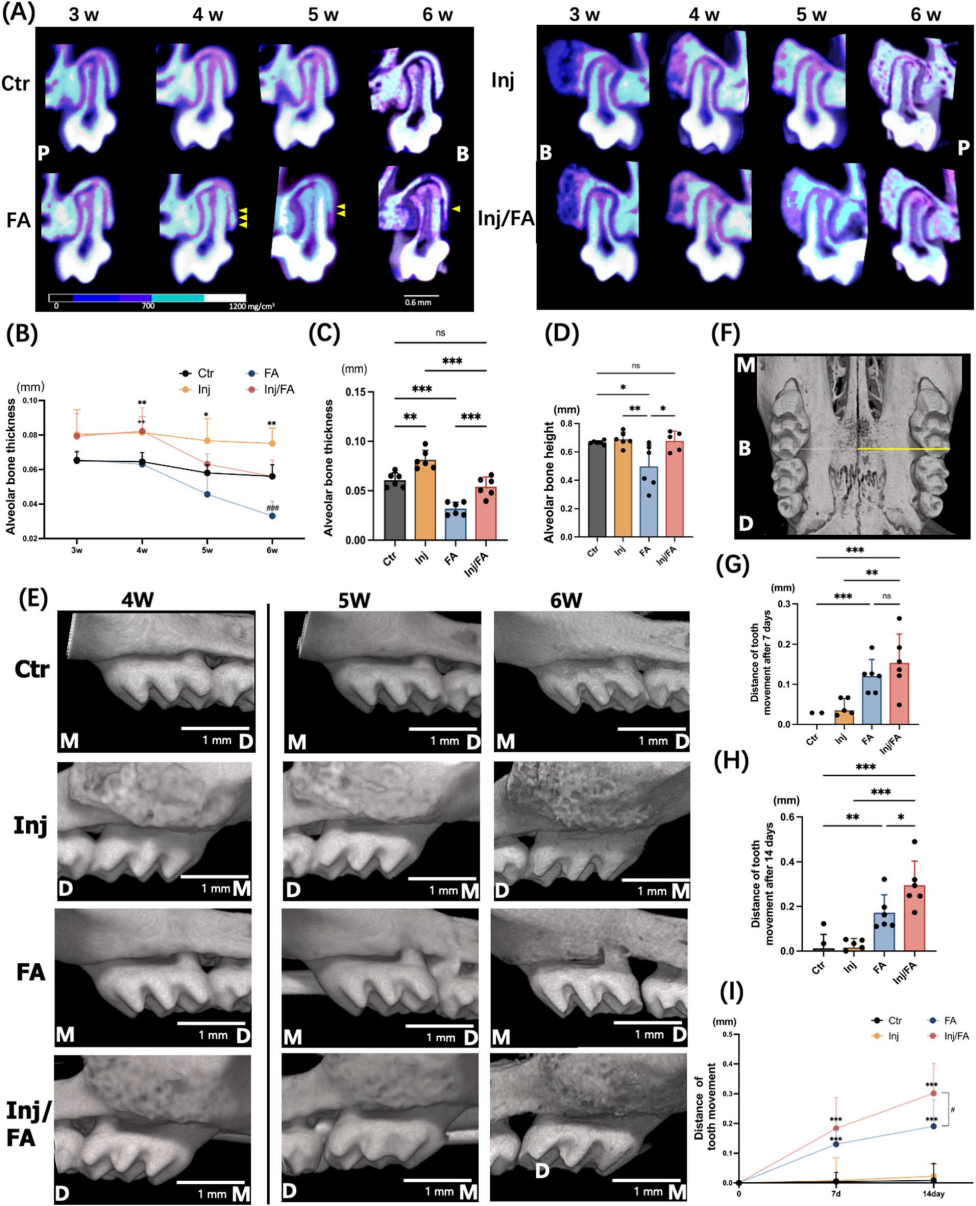
Changes in the in vivo μCT after injection and force application. (**A**) Changes in the bone mineral density (BMD) of the induced bone. Yellow arrow indicates the alveolar bone height. (**B**) Time-course of changes in buccal bone thickness from 3-week to 6-week. *p < 0.05, **p < 0.01: the Inj group versus the Ctr group; ^++^*p* < 0.01: the Inj/FA group versus the Ctr group; ^###^*p* < 0.001: the FA group versus the Ctr group. (**C**) Buccal bone thickness at week 6. (**D**) Alveolar bone height of each group after 2-week of force application. (**E**) Representative 3-dimensional reconstruction images of the buccal views of maxillary first molar. Scale bar = 1 mm. (**F**) The distances from distobuccal tip of M1 to mid-palatal suture (yellow line) were measured. (**G**,**H**) Measurement of tooth movement distance after 7- and 14-day of force application. (**I**) Time-course of changes in distance after force application. ^#^*p* < 0.05: the FA group versus the Inj/FA group; ***p < 0.001: the FA group and the Inj/FA group versus the Ctr group. *M* mesial, *D* distal, *P* palatal side, *B* buccal side.

**Figure 2:**
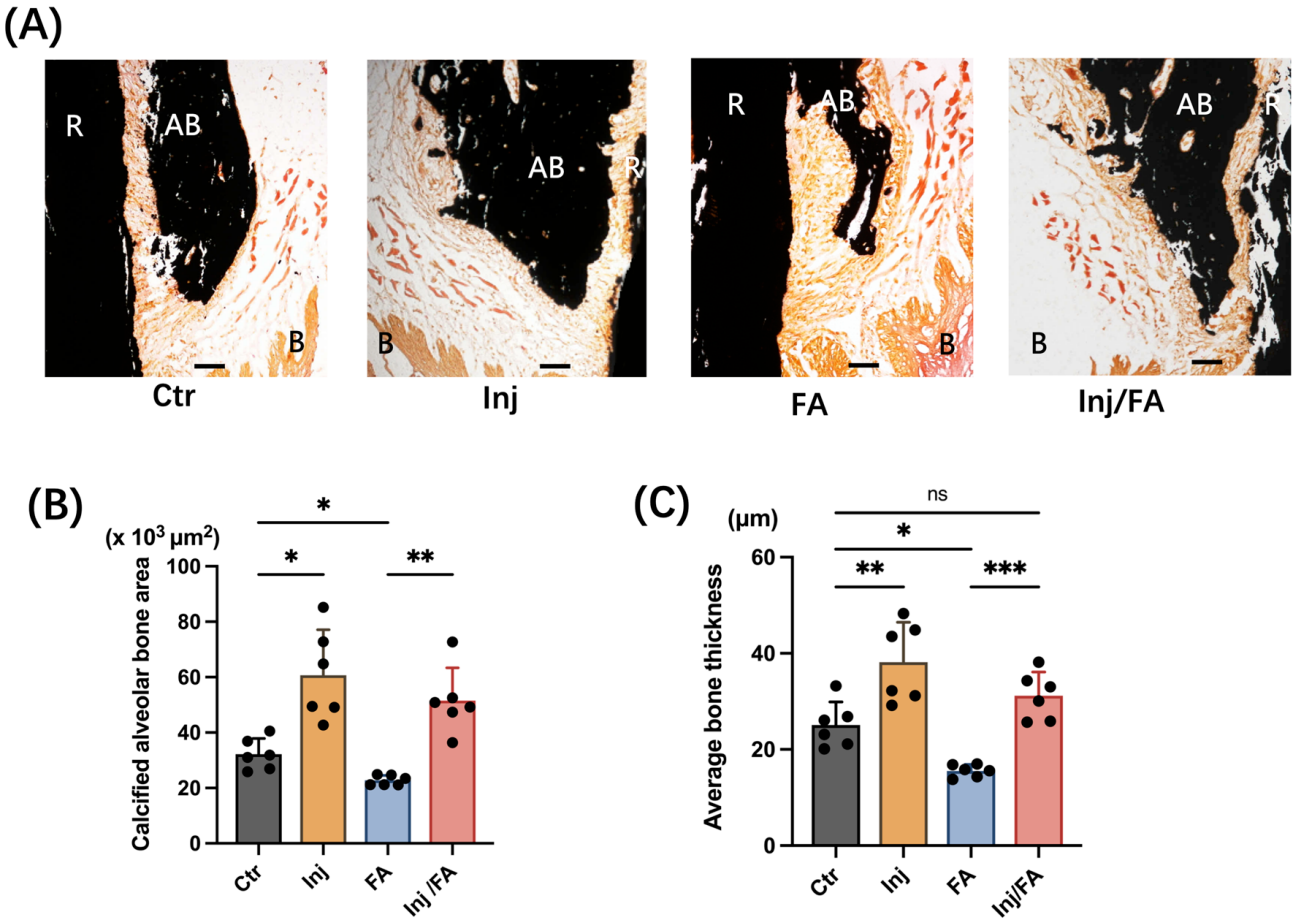
Calcified tissue measurements on the buccal alveolar bone. (**A**) Von Kossa-stained images of the alveolar bone at the distobuccal root (DBR). Scale bar = 50 μm. (**B**,**C**) Quantitative analysis of calcified bone of buccal alveolar bone. Normality was analyzed by the Shapiro–Wilk test. The comparisons among experimental groups were performed using ANOVA and Tukey HSD test. Values are expressed as the mean ± SD, *p < 0.05, **p < 0.01, ***p < 0.001, *ns* not significance, *AB* alveolar bone, *R* root, *B* buccal side.

Changes in the buccal alveolar bone were tracked for six weeks following anabolic injection. In vivo micro-focal computed tomography (μCT) images of the maxillary first molar (M1) at distobuccal root (DBR) site showed that the alveolar bone thickness and alveolar bone height of the Ctr group were at the same levels throughout the experiment, while the FA group showed continuous decrease in both alveolar bone thickness and alveolar bone height after force application (Fig. 1A). To confirm the μCT observations, quantitative analyses were performed. Alveolar bone thickness of the Ctr group showed no significant differences at weeks 5 and 6 compared to week 4. In the FA group, the alveolar bone at week 5 was 26.3% thinner than that at week 4. Furthermore, the alveolar bone thickness at week 6 showed a 42.1% decrease compared with that at week 4 (Fig. 1B). At week 6, the FA group was 46% lower in alveolar bone thickness compared to the Ctr group (Fig. 1C). Quantitative analyses of the alveolar bone height showed analogous results. Alveolar bone height in the FA group was significantly lower than that in the Ctr group at week 6 (Fig. 1D). The distance between the cementoenamel junction (CEJ) and the alveolar crest was significantly higher in the FA group than in the other groups (Supplementary Fig. 1). The three-dimensional (3D) μCT images of the M1s confirmed that no bone dehiscence was observed on buccal side of the alveolar bone before force application in all four groups at week 4. However, bone dehiscence was observed in the buccal alveolar bone at the DBR site of M1 in the FA group after seven days of force application and was aggravated in the following week (Fig. 1E). Consistent with the data from radiological analyses, Von Kossa staining showed that the FA group had 28% smaller calcified alveolar bone area and 27.8% thinner average alveolar bone than the Ctr group (Fig. 2A–C).

### Anabolic injection prevented force application induced bone dehiscence

The injections with gelatin hydrogel alone were performed firstly to confirm no bone augmentation at the site of injection, although we had already shown no osteoconductive effects of gelatin hydrogel itself^29,31^. As we expected, the injections of gelatin hydrogel alone could not induce apparent bone formation (Supplementary Fig. 2). On the other hand, with the injection of gelatin hydrogel, BMP-2 and OP3-4 peptide, the Inj and Inj/FA groups showed significantly thicker alveolar bones than the Ctr group at week 4 (Fig. 1A,E). The Injection groups continued to show a steady and thicker value in alveolar bone thickness compared to the other groups from weeks 4 to 6. In addition, the mesial-distal distance of the regenerated bone in injection groups shown in the 3D μCT images (Fig. 1E) presented similar as the mesial-distal distance of the yellow area obtained from the in vivo imaging system (IVIS) (Supplementary Fig. 3A), suggesting that the range of the scaffold delivery would be a guide for the size and the range of the regenerated bone. After force application, the Inj/FA group showed a continuous decrease in alveolar bone thickness (Fig. 1B). At week 6, the Inj/FA group kept a similar level of alveolar bone thickness as the Ctr group and was significantly thicker in alveolar bone thickness compared to the FA group (Fig. 1C). Additionally, in the first week after force application, the Inj/FA group showed a similar rate of change in alveolar bone thickness as the FA group. In the second week of force application, the decrease in alveolar bone thickness was alleviated in the Inj/FA group compared to the FA group (Supplementary Fig. 4). There were no significant differences in the alveolar bone height between the Ctr and Inj/FA groups. The Inj/FA group showed a similar level of alveolar bone height as the Ctr group and a higher value of alveolar bone height than the FA group (Fig. 1D). In addition, no significant differences were observed in the distance between the CEJ and alveolar crest between the Inj/FA and Ctr groups (Supplementary Fig. 1). The 3D μCT images did not show any dehiscence of the buccal alveolar bone in the Inj/FA group throughout the study period (Fig. 1E). Similar to the μCT analyses, the Inj/FA group showed no significant difference in the average bone thickness at week 6, but had a significantly larger calcified bone area compared to the Ctr group in von Kossa staining. The Inj/FA group also had a significantly larger calcified bone area and thicker bone than the FA group (Fig. 2A–C).

### Combination of anabolic injection and force application promoted bone turnover

Analysis of the 3D images revealed a continuous increase in the tooth movement distance in both the FA and Inj/FA groups after 7 and 14 days of force application (Fig. 1G–I). When comparing the FA and Inj/FA groups, the Inj/FA group showed a significantly greater tooth movement distance than the FA group after 14 days of force application (Fig. 1H).

**Figure 3:**
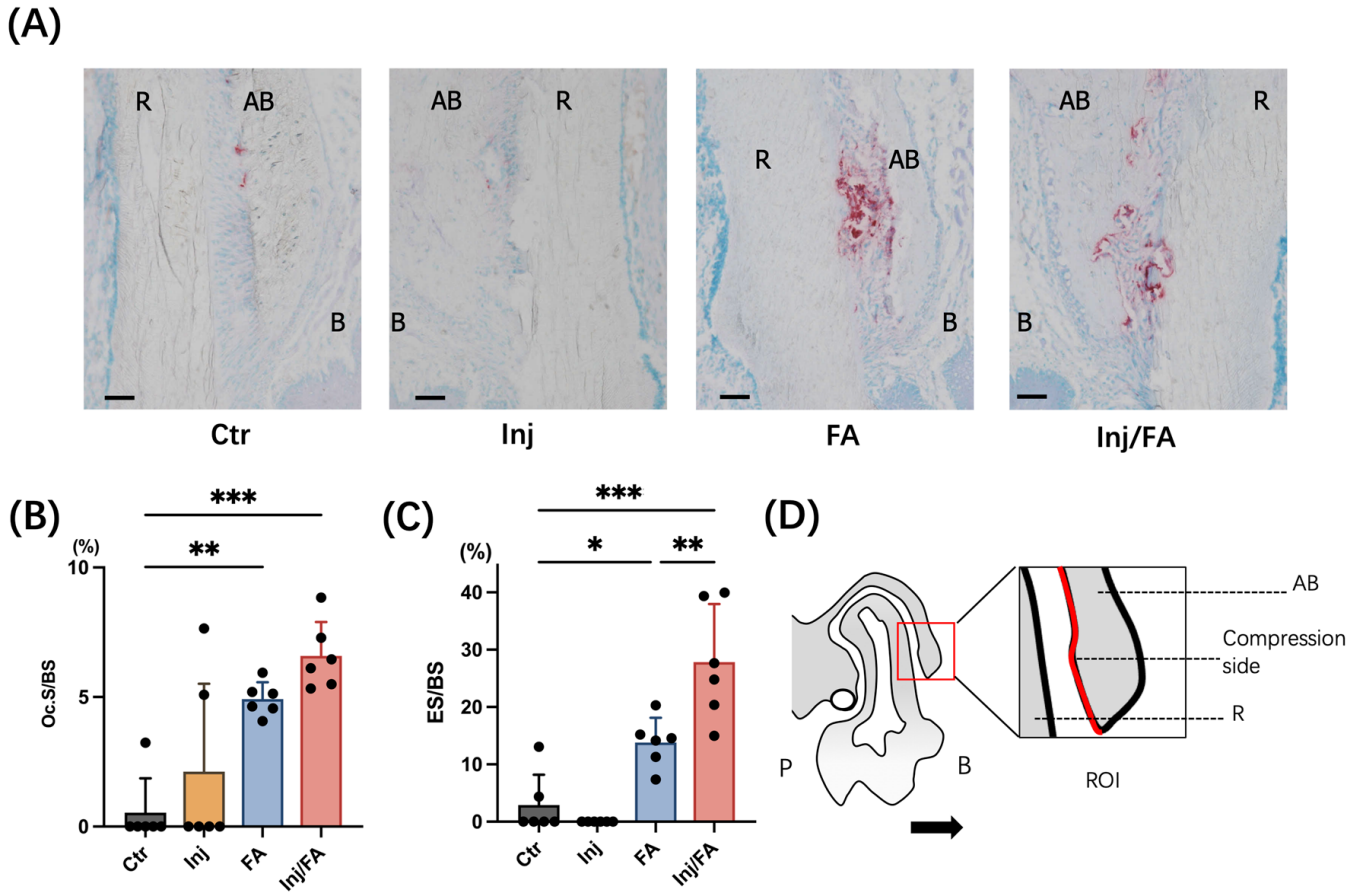
Osteoclastogenesis at the root-facing surface. (**A**) Images of TRAP staining after tooth movement. Force application induced osteoclastogenesis. Scale bar = 50 μm. (**B**) TRAP-positive cell surfaces were counted on the root-facing surface after tooth movement at DBR. (**C**) The eroded surface on the root-facing surface. (**D**) A schematic representation of the counting surface. Black arrow as the tooth movement direction; white circle as the wire. Values are expressed as the mean ± SD, *p < 0.05, **p < 0.01, ***p < 0.001. *AB* alveolar bone, *R* root, *P* palatal side, *B* buccal side.

**Figure 4:**
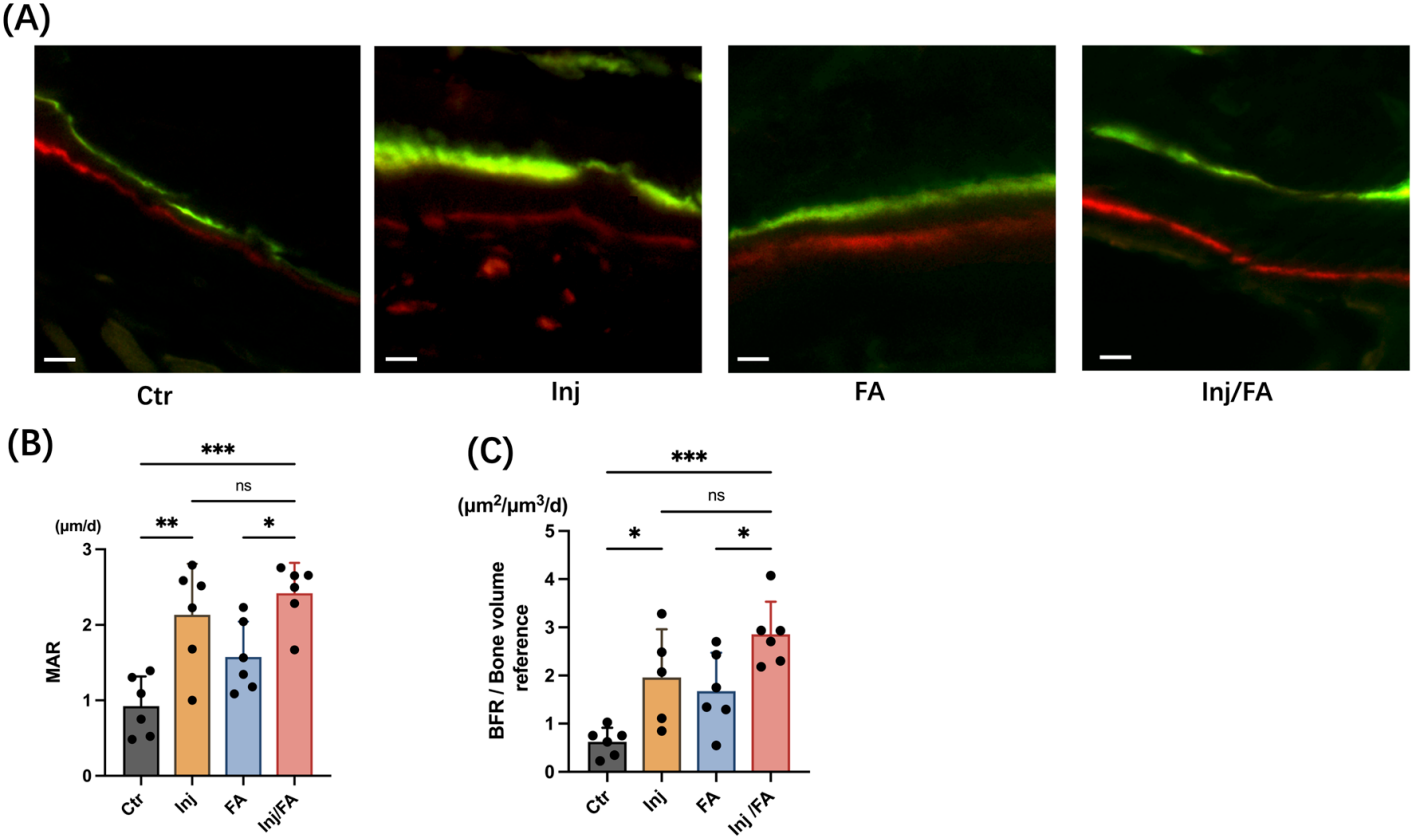
Bone formation activity at the buccal alveolar bone after injection and force application. (**A**) Fluorescence images of inter-labelled lines at the buccal alveolar bone. Green fluorescence: calcein administered on day 15 before euthanization; red fluorescence: alizarin administered on day 5 before euthanization. Scale bar = 10 μm. (**B**,**C**) Quantitative analysis of bone histomorphometry at DBR site, *MAR* mineral apposition rate, *BFR* MS × MAR/bone volume. Values are expressed as the mean ± SD, *p < 0.05, **p < 0.01, ***p < 0.001, *ns* not significance.

Further investigation was performed to clarify the bone remodelling activity in the local alveolar bone. Tartrate-resistant acid phosphatase (TRAP)-positive multinucleated osteoclasts appeared on the compression side of the alveolar bone after force application in both the FA and Inj/FA groups (Fig. 3A,D). Quantitative analyses of the osteoclast surface per bone surface (Oc.S/BS) and eroded surface per bone surface (ES/BS) confirmed these observations, and the FA and Inj/FA groups presented higher values in both Oc.S/BS and ES/BS than the Ctr group (Fig. 3B,C). Furthermore, the ES/BS ratio was significantly higher in the Inj/FA group than in the FA group. Fluorescent labelling images revealed that the mineral apposition rate (MAR), which represents osteoblast activity, was 132% higher in the Inj group and 198% higher in the Inj/FA group than in the Ctr group. The MAR of the Inj/FA group was 54% higher than that of the FA group (Fig. 4A,B). Similarly, the bone formation rate (BFR) showed prominent bone formation activity in both the Inj and Inj/FA groups compared to the Ctr group during the 14 days of force application (Fig. 4C). Meanwhile, no significant difference was observed in either MAR or BFR between the Inj and Inj/FA groups.

## Discussion

In this study, we developed a mouse model of FA-induced bone dehiscence. Bone dehiscence is commonly detected after tooth movement in a narrow alveolar ridge, which jeopardises long-term stability. In the current clinical treatment, no standard treatment methods have been developed to prevent bone dehiscence induced by tooth movement^33^. Therefore, it is crucial to explore alternative approaches for preventing bone dehiscence. Additionally, assessing the alveolar ridge for bone dehiscence during dental treatment is essential. Bone dehiscence can be defined as an increase in the distance between the CEJ and the buccal or lingual alveolar bone crest^34^. In this study, bone dehiscence appeared on the buccal side of M1 after force application (Fig. 1E). The distances between the CEJ and the alveolar bone crest were significantly greater in the FA group than in the other groups (Supplementary Fig. 1). In addition, the development of bone dehiscence is commonly related to alveolar bone height and thickness^35,36^. Our data showed that the alveolar bone height and thickness of the FA group were also significantly lower (Fig. 1B–D). Our results confirmed the presence of bone dehiscence in the FA group. Thus, an FA-induced bone dehiscence mouse model was developed to elucidate the bone response to mechanical force in a narrow alveolar ridge.

A previous study showed that the bone mineral density (BMD) of the induced bone increased continuously within 8 weeks after the anabolic injection on the palatal side^32^. In our study, a prophylactic injection resulted in the BMD of the induced bone increasing gradually at the buccal side during the six-week period (Fig. 1A). Bone augmentation is achieved by injecting anabolic bone reagents into the alveolar bone, leading to thicker alveolar bone for tooth movement and expanding tooth movement limitations. Since no osteoconductive effects of gelatin hydrogel was shown (Supplementary Fig. 2), it was also confirmed that the outcome of this study is caused by the effect of BMP-2 and OP3-4 peptide, not by the gelatin hydrogel scaffold. It is known that the rate of tooth movement decreases when the tooth is moved into a region with reduced alveolar bone^37^. Thus, it is possible that preliminary bone formation after injecting anabolic reagents in the alveolar ridge with anatomic restrictions allows tooth movement without delay. On the other hand, no bone dehiscence was observed in the Inj/FA group until the end of force application, and both the alveolar bone thickness and alveolar bone height in the Inj/FA group remained at a level similar to that in the Ctr group after applying force. Surprisingly, in the Inj/FA group, the rate of change in alveolar bone thickness began to decrease at week 5, whereas that in the FA group increased continuously (Supplementary Fig. 4). These results suggest that the injection of anabolic bone reagents can protect the narrow alveolar ridge from the effects of force application.

The rate of tooth movement in a narrow alveolar ridge is inhibited compared to when the teeth are moved to a location with sufficient bone^4^. Given that in our study force application led to bone dehiscence (Fig. 1E), the alveolar ridge to which the tooth moved due to force application was thought to be narrow, and the speed of tooth movement in the FA group should have been inhibited. In contrast, a tooth with sufficient alveolar bone width moves faster than a tooth with a thin alveolar bone^38^. Due to the augmentation of the alveolar bone achieved by anabolic injection in the Inj/FA group (Fig. 1A), the shortening of the tooth movement period was supposed to have taken place. In this study, our results confirmed the above-mentioned hypothesis, revealing that the tooth movement distance was greater in the Inj/FA group within two weeks of force application than in the FA group (Fig. 1H).

Shortening the duration of tooth movement treatment has been reported to be associated with a decrease in certain side effects, such as oral hygiene-related problems, gingival recession, and root resorption^39^. So far, several approaches have been utilised to shorten tooth movement treatment. Recently, a minimally invasive injection method using platelet-rich plasma (PRP) was reported to shorten the treatment period^40^. A study by Alomari et al. showed that bone dehiscence and fenestrations were not prevented by orthodontic treatment with PRP injection^41^. However, our findings suggested that the use of the anabolic injection could shorten the tooth movement period without causing bone dehiscence during tooth movement.

We then clarified the morphological and biological changes in the alveolar bone after applying force to the area where bone augmentation occurred. Bone histomorphometric analyses showed higher bone remodelling activity in the Inj/FA group than in the FA group (Figs. 3 and 4). ES/BS, a bone resorption parameter indicating osteoclast activity, was significantly higher in the Inj/FA group than in the FA group. As for the bone formation activities, dynamic bone histomorphometric analyses using fluorescent-labelled images showed a higher value of MAR and BFR in the Inj/FA group compared to the FA group. This implied that the combination of anabolic injection and force application led to a higher level of bone formation. These results suggested that a higher bone remodelling activity was induced by anabolic injection in the local alveolar bone. This could explain the faster tooth movement in the Inj/FA group than in the FA group.

Our study investigated a minimally invasive injection method of BMP-2 and OP3-4 peptide, and showed that the anabolic injection method could achieve alveolar ridge augmentation and expand the range of tooth movement. Additionally, the combination of anabolic injection and force application led to further tooth movement without evident bone dehiscence. In conclusion, we developed the first FA-induced bone dehiscence mouse model and showed that a minimally invasive anabolic injection method could prevent bone dehiscence induced by force application. Thus, this method has a high potential to facilitate orthodontic treatment for patients with anatomic limitations and might be useful in treating periodontal disease, prosthetic treatment with dentures, dental implants, and tooth transplantation accompanied by missing teeth.

## Methods

### Animals

Twelve seven-week-old C57BL/6J male mice bred by NIPPON CLEA (Tokyo, Japan) were used. All mice were given a week to adapt to a 12-h dark/light cycle under constant temperature (22 ± 1 °C) with ad libitum access to food and water. This study was approved by the Institutional Animal Care and Use Committee of Tokyo Medical and Dental University (TMDU) (approval number: 2021-259C, 2021-135C). We can confirm that all methods were performed in accordance with the relevant guidelines and regulations. All animal experiments were conducted in strict accordance with the Animal Research Reporting of In Vivo Experiments (ARRIVE) guidelines and regulations for care and use of laboratory animals.

### Experimental design and surgical procedure

**Figure 5:**
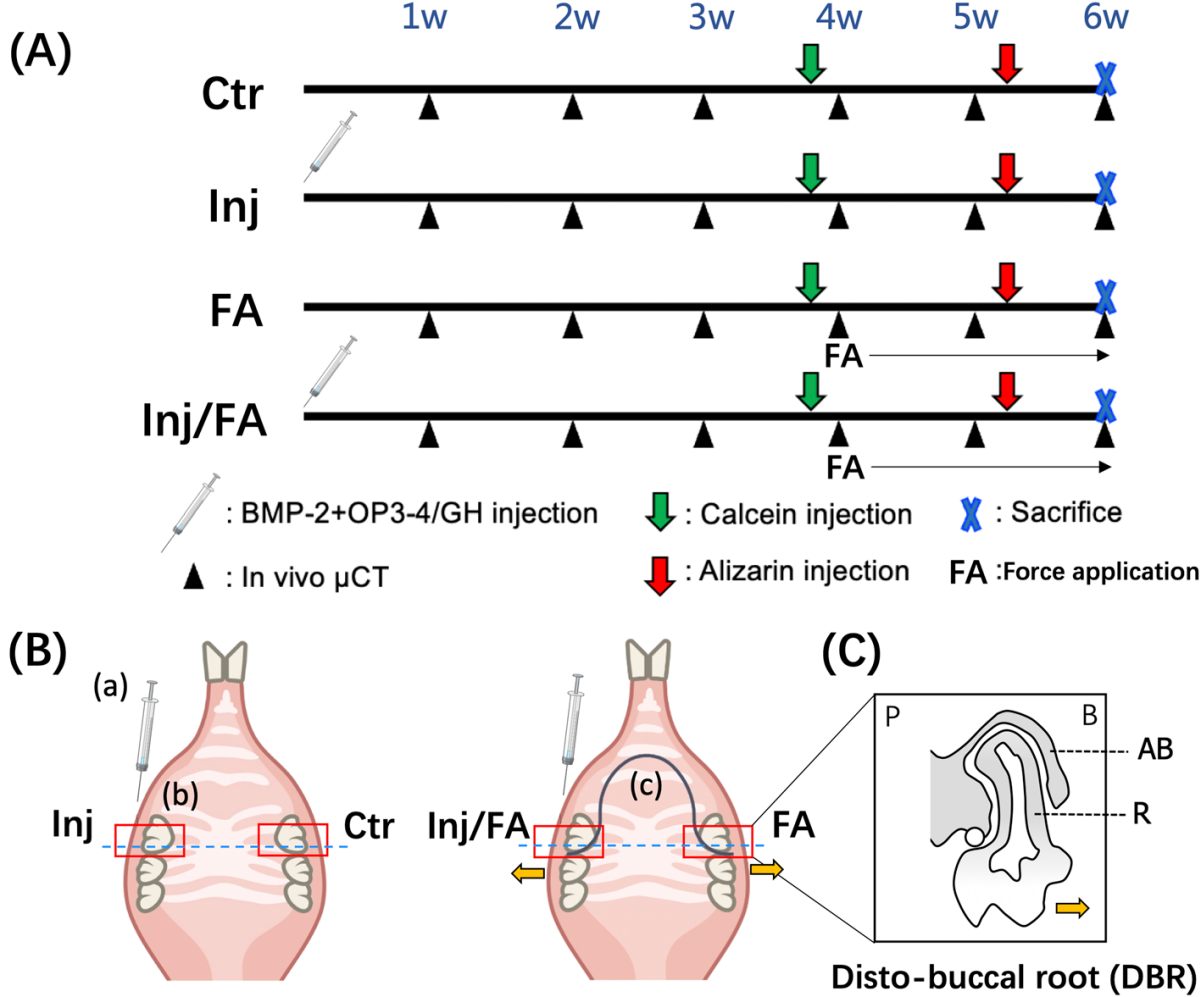
Experimental protocol. (**A**) Timeline diagrams of the design. The schedule for the injection of bone anabolic reagents, force application and fluorescent labelling. (**B**) A schematic representation of the experimental groups: (a) Hamilton syringe; (b) Upper first molar; (c) Tooth movement wire. (**C**) A schematic representation of the section for analysis. *AB* alveolar bone, *R* root, *P* palatal side, *B* buccal side. The yellow arrow indicates the tooth movement direction.

Three mice were used in a preliminary study to investigate the effects of gelatin hydrogel itself on bone formation (granule size of gelatin hydrogel: 50 µm-diameter and 250 µm-length, the Japan Wool Textile Co., Ltd., Osaka, Japan). These three mice received an injection of gelatin hydrogel alone on the left side of the maxilla bone and were sacrificed at four weeks after the injections. Micro CT images were obtained at the end of the experiment. Then a mixture of gelatin hydrogel fibre scaffolds, BMP-2 (Bioventus LLC, Durham, NC, USA), and anabolic peptide (OP3-4; ABclonal Biotechnology, Woburn, MA, USA) was prepared for injection. The ratio of BMP-2 and OP3-4 was at a ratio described in a previous study^31^, BMP-2 (1 μg in 1 μl LF6 buffer) was mixed with gelatin hydrogel (0.6 mg) with OP3-4 (0.66 mg, dissolved in 5 μl dimethyl sulfoxide), and the mixture was incubated at room temperature for 4 h. A total of 12 eight-week-old male C57BL/6J mice were subcutaneously anaesthetized as previously described^32^. All the mice received a 2-μl submucosal injection on the buccal side of the right maxillary M1 using a 26-gauge, 15° tip Hamilton needle attached to a 25-μl Hamilton syringe (Fig. 5A). Due to the delicate nature of the mucosal tissue, the injection site where the needle was inserted had to be placed approximately 2.0 mm proximal from bone formation site, namely the buccal alveolar bone of M1. Additionally, six mice were injected with gelatin hydrogel impregnated with alizarin on the right buccal side of maxillary first molar, and the left side was injected with gelatin hydrogel without alizarin. The maxillae were excised and then obtained a fluorescent image using in vivo imaging system (IVIS) (IVIS Lumina XRMS Series III, Revvity Inc., Waltham, MA, USA) to evaluate the distribution of the gelatin hydrogel injected to maxilla at 4 h and 3 days after the injections. Excitation and emission filters was 560/620 nm. Then undecalcified sections were made (Supplementary Fig. 5).

Four weeks after the injection, a U-shaped tooth movement appliance was applied to 50% of the randomly selected mice. The appliance was made with a nickel-titanium round alloy wire of 0.2 mm diameter and 10 mm length (Furukawa Electric Co. Ltd., Tokyo, Japan). The wire was bent and adjusted using a dial tension gauge (DTN-10; TECLOCK, Tokyo, Japan) and bonded to the palatal surfaces of the right and left maxillary M1s using a light-curing composite resin (G-FIX; GC, Tokyo, Japan) to exert a 10 cN reciprocal force toward the buccal side (Fig. 5. Direction shown as yellow arrow). All appliances were checked daily throughout the period of force application. Since the applied force was still active until day 14, we did not reactivate the wire (Supplementary Fig. 6).

Then, the mice were randomly divided into four groups (n = 6 for each group): the control (Ctr) group , the injection (Inj) group , the force application (FA) group, and the injected group with force application (Inj/FA) group (Fig. 5B). The sample size was determined using power analysis based on data from our previous pilot studies. On days 27 and 37, the mice were subcutaneously injected with the following fluorescent reagents: calcein (20 mg/kg; Sigma-Aldrich Co., Ltd., St. Louis, MO, USA) and alizarin complexone (20 mg/kg; Dojindo, Kumamoto, Japan), and euthanised by cervical dislocation under anaesthesia at week 6. The cranial bones were then dissected and fixed in phosphate-buffered saline (PBS)-based formaldehyde solution fixative (pH 7.4) for 18 h at 4 °C under constant shaking motion. All the samples were washed and stored in PBS for radiological and histological analyses.

### Radiological assessment

After the second week after injection, in vivo μCT (R_mCT2 SPMD, Rigaku, Tokyo, Japan) imaging was performed weekly (90 kV, 160 μA, 10-mm field of view). 3D reconstruction images of the maxillae were captured and reconstructed using image analysis software (TRI/3D-BON; Ratoc System Engineering, Tokyo, Japan), and arranged such that the occlusal plane was parallel to the floor and balanced between the left and right planes. Two-dimensional images of the sagittal planes were first defined as the plane passing through the centre of the mesial root canal and the DBR canal, and then the coronal views were defined as the plane passing through the DBR canal (Supplementary Fig. 7). Coronal sections were used to observe BMD changes (Fig. 1A). Then the same coronal views of the image data were blinded, evaluated to measure the buccal alveolar bone thickness at the DBR site, region of interest (ROI) of 500 × 500 µm was set from the CEJ. Alveolar bone thickness was analysed using ImageJ software (version 1.50 i; NIH, Bethesda, MD, USA). The change in alveolar bone thickness was calculated by subtracting the thickness at week 4 from week 5, and week 5 from week 6. Then, the weekly thickness changes were divided into seven days to calculate the rate of change after force application. In the same coronal view, the buccal alveolar bone height was measured from the most lingual aspect of the buccal bone at the level of the apex to the beginning of the high BMD (> 690 mg/cm^3^). The distance of tooth movement was calculated by subtracting the distance between the midpalatal suture and the distobuccal cusp of M1 (Fig. 1F) at the start of force application (week 4) from 7-day force application (week 5) to 14-day force application (week 6) after force application.

### Histological assessment and bone histomorphometry

The maxillae were cut just anterior to the M1 under a constant flow of water. The samples were then embedded in SCEM compound (Section-Lab Co. Ltd, Hiroshima, Japan) and frozen into blocks at − 100 °C in a freezing system (UT2000F; Leica Microsystems, Wetzlar, Germany). Two- and 5-μm-thickness slices of undecalcified frozen sections at DBR sites were prepared using a cryomicrotome (CM3050sIV; Leica Biosystems, Nussloch, Germany) according to Kawamoto's method with adhesive film (Cryofilm type 2C(9), Section-Lab Co. Ltd), as described elsewhere^42^. Two-μm slices were stained according to the von Kossa staining method to detect the calcified tissue. Osteoclasts were identified using TRAP staining with methyl green counterstaining^32^. Bone histomorphometry was performed at an ROI of 500 × 500 µm from the crest of the alveolar bone at the DBR section. Osteoclasts were defined as TRAP-positive multinucleated cells with at least two nuclei on the bone surface, which was measured as the root-facing surface. Oc. S/BS and ES/BS were measured and calculated.

Fluorescence-labelled images were used to analyse bone formation activity in the alveolar bone. All sections (5-μm) were blinded, and viewed under a fluorescence microscope (FSX100; Olympus, Tokyo, Japan). The MAR was calculated by measuring the inter-labelled distance between a calcein-labelled line (green) and an alizarin-labelled line (red) at 10-day intervals. The area reference (BFR) was calculated by multiplying the mineralising surface (MS) by the MAR and then dividing by the bone volume^43,44^. Bone histomorphometric analyses were performed using ImageJ software.

### Statistical analysis

All mice were used for the measurements, and no mice were excluded from data analysis. All statistical analyses were performed using the SPSS ver. 25.0. Multiple intergroup comparisons were performed using one-way analysis of variance (ANOVA), followed by Tukey’s post-hoc test. Normality was analysed using the Shapiro–Wilk test. When a significant F ratio was identified, the groups were compared using non-parametric analyses using the Kruskal–Walli’s test. The calculated *p*-values were adjusted using the Bonferroni correction. Statistical significance was set at *p* < 0.05 indicating statistical significance. All data are presented as means ± standard deviation (SD).

### Supplementary Information

Supplementary Figures.

## Data Availability

All data associated with this study are provided within the main manuscript or a supplementary information file.
