# Supplementary material for: Prevention of bone dehiscence associated with orthodontic tooth movement by prophylactic injection of bone anabolic agents in mice
Source: Sci Rep. 2024 Jul 8;14:15749. doi: 10.1038/s41598-024-66617-6 (PMC11231170; doi:10.1038/s41598-024-66617-6)
Supplement: Supplementary file 1 — Supplementary Figures. [file 41598_2024_66617_MOESM1_ESM.docx]

**Injectable Bone Anabolic Reagents Prevent Bone Dehiscence by Tooth Movement**

**J. Qi^1,2^, Y. Matsumoto^1*^, C. Xie^2,3^, F. Rashed^2,4^, T. Ono^1^ and K. Aoki^2^**


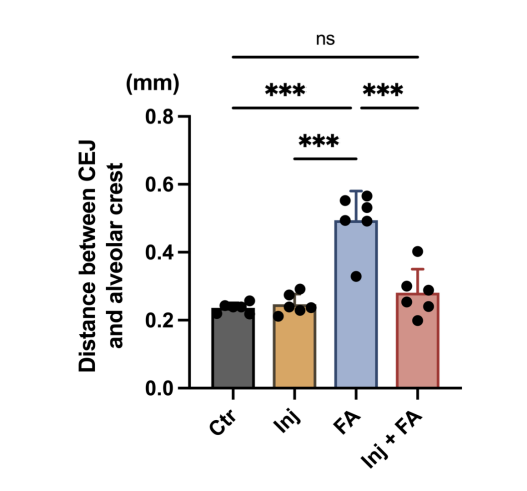


**Supplementary Figure 1.** Distance between the cement-enamel junction (CEJ) and alveolar crest. The distance was taken from the CEJ to the beginning of the high BMD on the same coronal views as ABH. Values are expressed as the mean ± SD, ****p* < 0.001, ns: not significance.


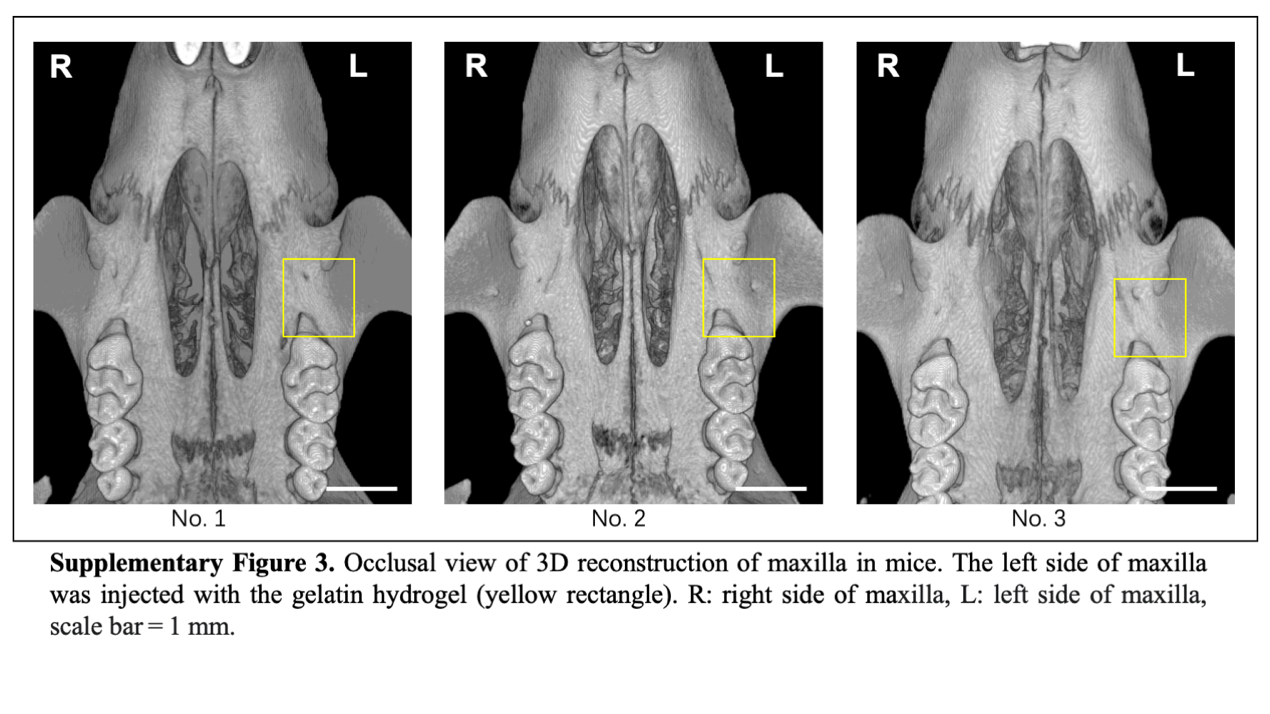


**Supplementary Figure 2.** Occlusal view of 3D reconstruction of maxilla in mice. The left side of maxilla was injected with gelatin hydrogel (yellow rectangle). No treatment for the right side of maxilla. R: right side of maxilla, L: left side of maxilla, scale bar = 1 mm.


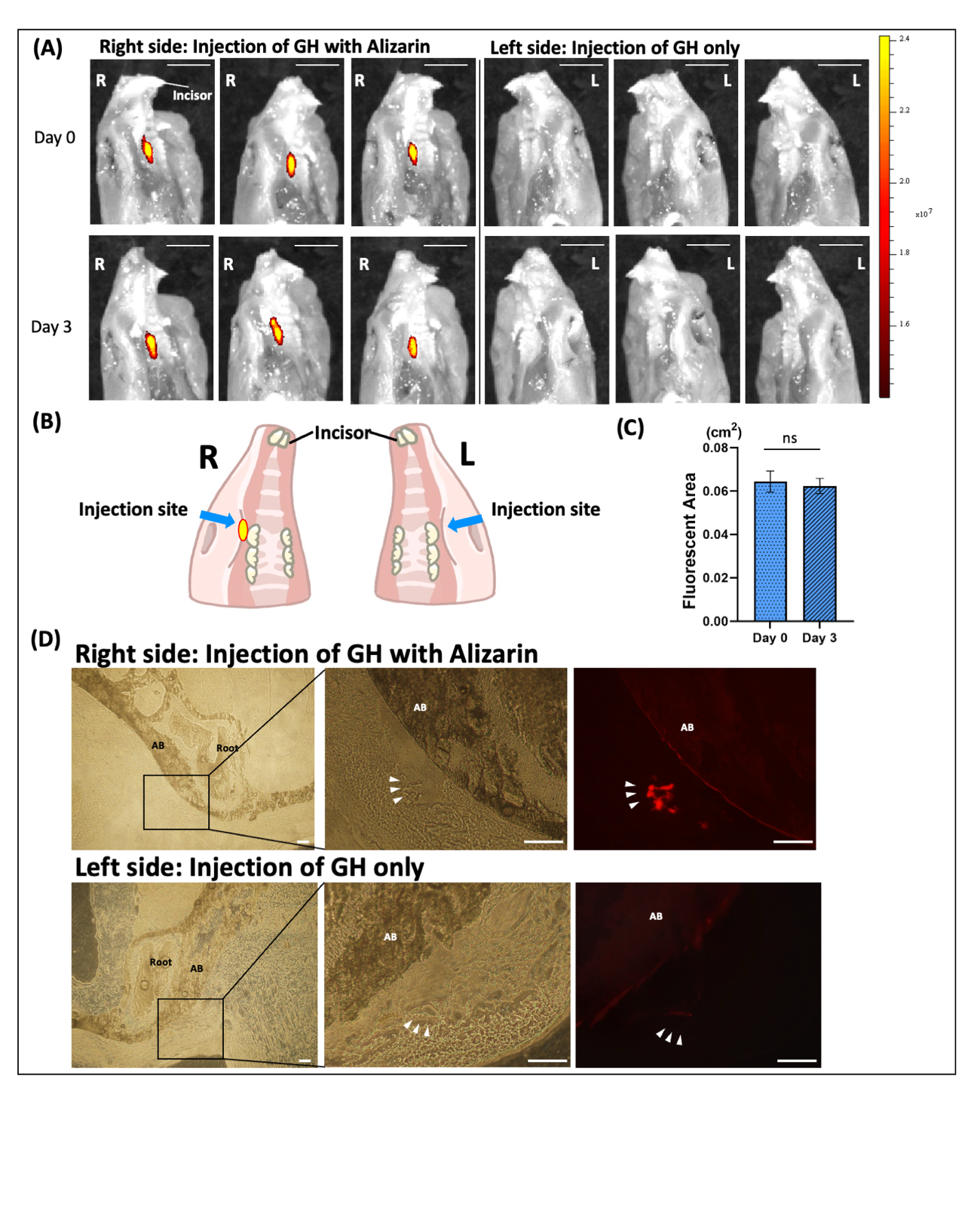
 **Supplementary Figure 3.** **Distribution of the gelatin hydrogel after injections to the buccal alveolar bone site. (A)** Ex vivo IVIS images of the dissected maxillae on day 0 and 3. **(B)** A schematic representation of the IVIS images for right and left side image. The yellow area represents an alizarin-positive area. R: right side; L: left side. **(C)** Statistic analyses of fluorescence-labelled area at each time-point. ns: no significant difference. Scale bar = 5 mm. **(D)** Fluorescent images of the alveolar bone. White arrow indicates gelatin hydrogel. Scale bar = 100 μm. GH: gelatin hydrogel.

**
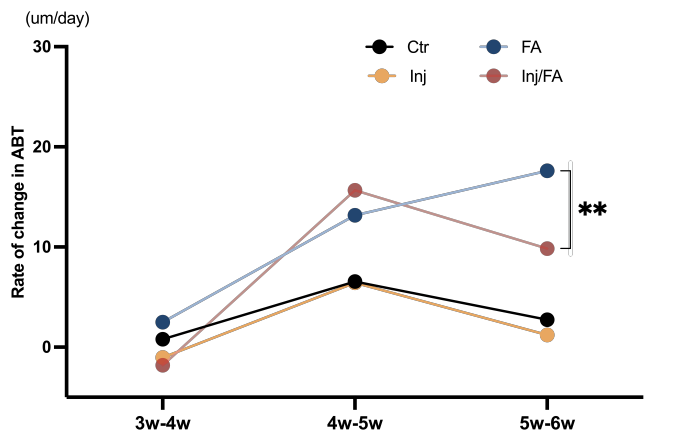
**

**Supplementary Figure 4.** Time-course for rate of changes in alveolar bone thickness (ABT). ***p* < 0.01: the Inj group versus the Inj/FA group.


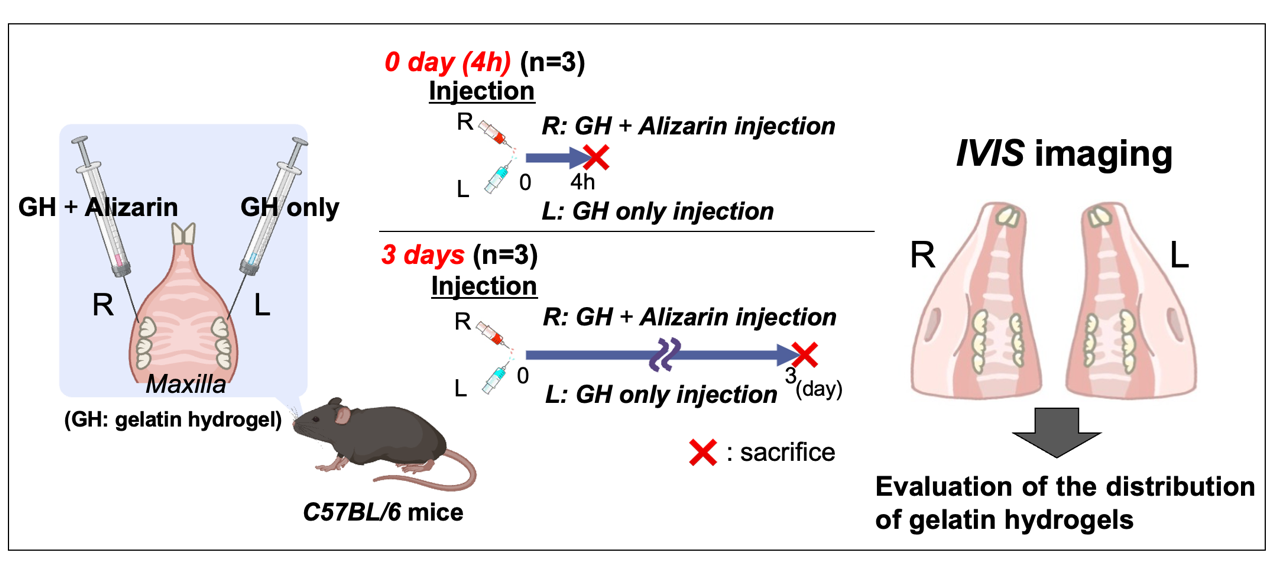


**Supplementary Figure 5:** Experimental protocol to clarify the distribution of the gelatin hydrogel after the injection to the mouse maxilla (buccal alveolar bone site).


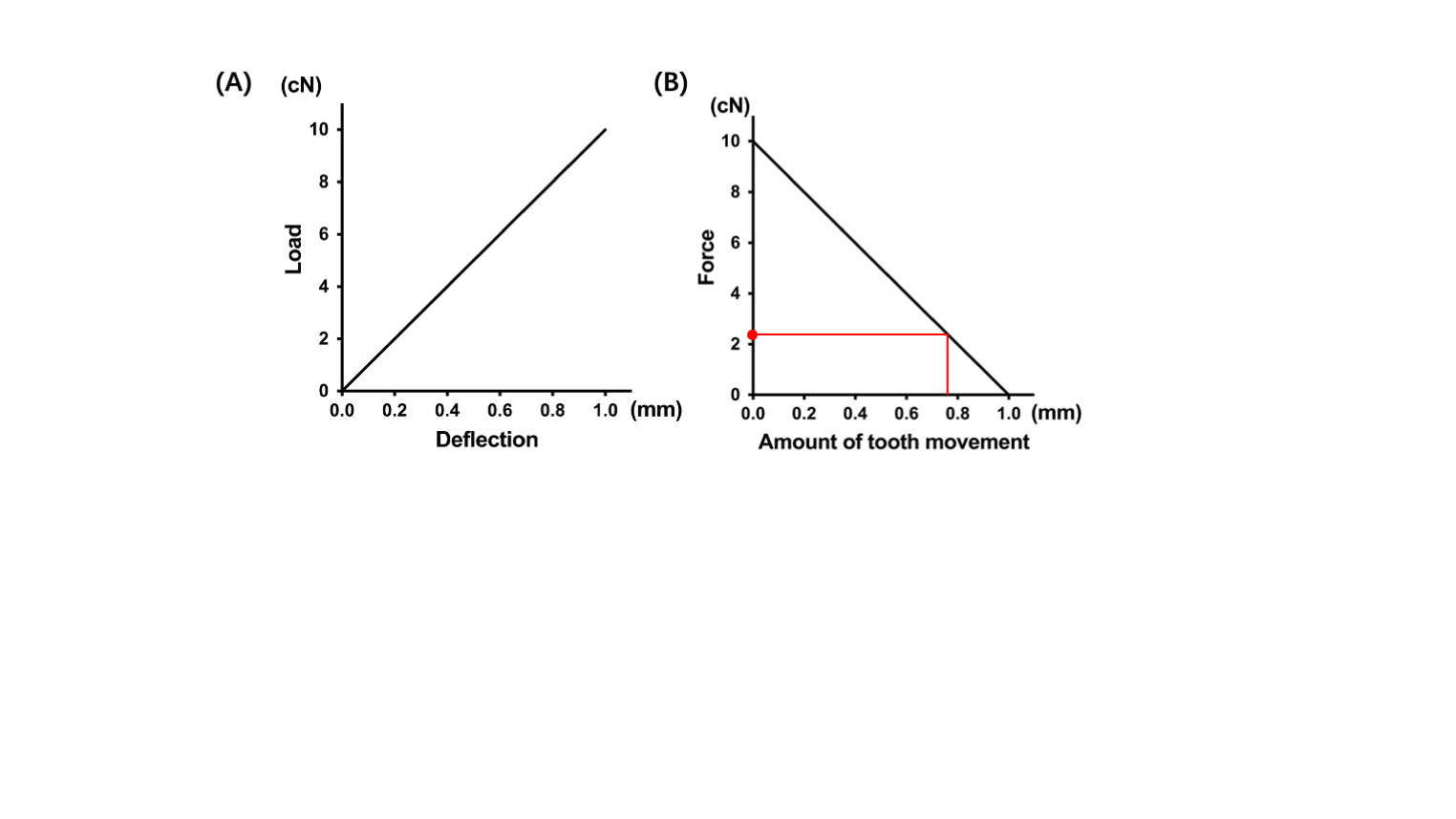


**Supplementary Figure 6.** **(A)** The load-deflection curve of the Ni-Ti alloy round wire used in this model. **(B)** The force remained after tooth movement. The red line refers to the minimum force remained at day 14.


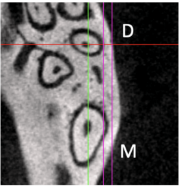


**Supplementary Figure 7.** Two-dimensional images of the coronal views were adjusted by sagittal planes, which passing through the center of the mesial root canal and distobuccal root (DBR) canal. Coronal views as the red line plane. M: mesial. D: distal.
